# Welding Characteristics of Medium Titanium Plates with Autogenous Laser Welding and Narrow-Gap Laser Filling Welding Modes

**DOI:** 10.3390/ma17194722

**Published:** 2024-09-26

**Authors:** Junzhao Li, Hang Yu, Xin Yin, Bin Kong, Kai Wen, Qingjie Sun, Bingfeng Wang, Xianshan Zeng

**Affiliations:** 1 School of Materials Science and Engineering, Central South University, Changsha 410083, China; 18816311219@163.com; 2 Hunan Xiangtou Goldsky Titanium Metal Co., Ltd., Changsha 410083, China; 3 Hunan Xiangtou Goldsky New Materials Co., Ltd., Yiyang 413000, China; 4 Shandong Provincial Key Laboratory of Special Welding Technology, Harbin Institute of Technology at Weihai, Weihai 264209, China

**Keywords:** pure titanium, medium plate, autogenous laser welding, narrow-gap laser welding

## Abstract

Titanium and titanium alloys with a medium thickness of 5 to 12 mm are widely used for ocean platforms, military equipment and in other fields because of their light weight, appropriate strength and corrosion resistance. In this study, autogenous laser welding and narrow-gap laser welding processes were researched and compared, and the welding characteristics, weld microstructure and joint strength were analyzed. The results showed that autogenous laser welding had higher efficiency, narrower weld width and higher microstructure uniformity. Autogenous laser welding can achieve the single pass weld penetration at laser keyhole mode. The weld width of narrow-gap laser welded joint was 12.5 mm, which was nearly three times than that of autogenous laser welding. The grain size of autogenous laser welding was obviously smaller and more uniform in depth than that of narrow-gap laser welding. In the weld zone, the coarse columnar α grains grew from the fusion line, while in the heat-affected zone, equiaxed α grains with needle and sawtooth α morphologies were presented. The microhardness of the heat-affected zone was higher than in the weld zone and the base metal due to the denser needle microstructure. The tensile samples all fractured at the base metal, indicating the welded joint strength efficiency was greater than 1.

## 1. Introduction

Titanium and titanium alloys are widely used as important structural metals in aerospace, ocean engineering and other frontier fields because of their high strength, light weight, good corrosion resistance, high heat resistance and good weldability [1]. In the connection process of titanium and titanium alloys, welding has the advantages of effective utilization of materials, lower cost, short manufacturing cycle and avoiding potential corrosion [2]. Nowadays, welding has shown a trend of gradually replacing traditional connection methods such as riveting, and has become the core manufacturing technology of important structural parts in various fields [3]. At present, the common welding processes of titanium and titanium alloys mainly include laser welding, electron beam welding, tungsten inter gas welding (TIG) and metal inert gas welding (MIG). Electron beam and laser welding have the advantages of large heat source energy density, large weld depth and narrow width, fast welding speed and high welding efficiency when welding medium and thick plates with thickness of 5 to 120 mm [4,5]. Among these, the laser autogenous welding process can achieve effective welds with a thickness of 5 to 20 mm. However, thicker plates tend to be welded via the narrow-gap filling welding process with multiple layers.

For the welding of medium plate, the laser keyhole welding mode with high power is desired to increase weld penetration. However, some researchers have found that the welding process becomes unstable and weld defects appear as the power increases. Laser welding porosity mainly exists in the fusion zone with two morphologies. The metallurgical pores with small size and a smooth inside wall are caused by hydrogen accumulation, while the unstable laser keyhole leads to process pores with an irregular shape. The solubility of hydrogen in titanium decreases with the increase in temperature and has a sudden change at the melting point. The directional diffusion of hydrogen is caused by local melting, solidification, phase change and uneven temperature in the welding process. Therefore, the hydrogen porosities tend to gather in the fusion line zone in a chain form. Hydrogen porosities can be eliminated by optimizing the shielding device and regulating welding parameters [6,7,8]. Huang et al. [7] studied the formation mechanism of porosity and considered that the porosity can be controlled by increasing the existing time of the high-temperature molten pool even though the hydrogen content was high in the weld. As for process porosities, Chang et al. [8] suggested that in keyhole laser welding, a porosity formation mechanism was closely related with the turbulent fluid flow behind the keyhole. In the full welding penetration condition, it has been proven that decreasing the Reynolds number of molten flow should be an effective way to reduce the porosity of laser welds. Pang et al. [6] revealed that the pore number and weld morphology were relevant to the keyhole depth oscillation. The welding speed is a crucial parameter that affects the stability of the molten pool. Rapid cooling can lead to a substantial decrease in vapor pressure inside the keyhole tip, which was suggested to be the mechanism by which shielding gas enters into the bubbles. For multi-layer narrow-gap laser welding of titanium alloy, the laser heat conduction mode is usually used to melt filler wire and base metal. During narrow-gap laser welding, the incomplete fusion of the sidewall and filling layer is the crucial defect, which leads to local stress concentration and reduces bearing area of the welded joint. In order to solve this problem, new technologies such as beam oscillation and double beam heat source, have been introduced to optimize heat distribution [9,10,11,12]. Among this, beam oscillation laser welding can reset the heat source energy into the groove sidewalls to increase sidewall penetration, just like the effect of swing MAG arc welding and magnetic-controlled TIG welding for narrow-gap welding. Furthermore, some researchers have indicated that the fluidity of molten metal in the groove was also regulated by beam oscillation to influence weld bead formation and accelerate the escape of gas.

Titanium medium plate is widely used for offshore platform structures, while the connection of these plates is the main problem to be solved. This study aimed to research the characteristics of laser autogenous welding and narrow-gap laser filling welding of titanium medium plate. The welding parameters and microstructure evolution in various zones with heating cycles were compared and evaluated for the guidance of practical application.

## 2. Materials and Methods

**Figure 1:**
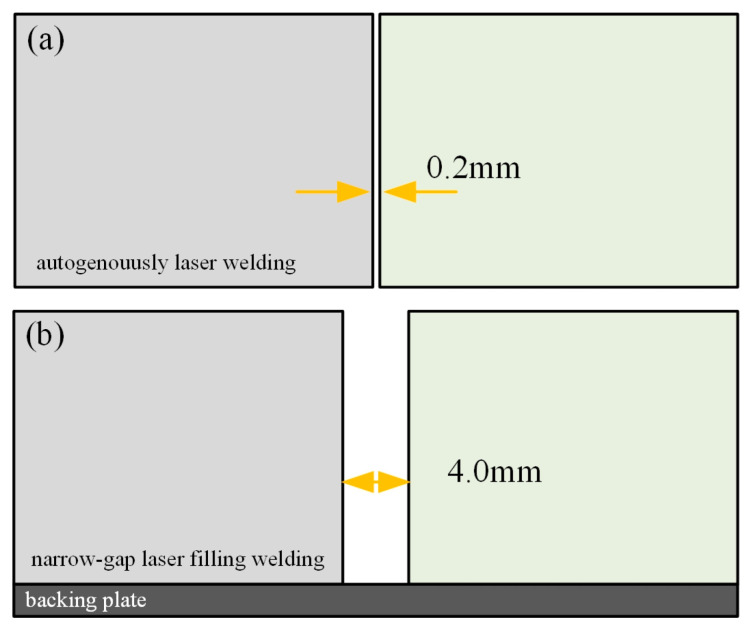
Schematic diagram of the welding process: (**a**) autogenously laser welding; (**b**) narrow-gap laser filling welding.

The base material used in this experiment was pure titanium Gr.2 plate with a thickness of 7 mm. The tensile strength and yield strength of the base metal were 430 Mpa and 265 Mpa, respectively. Sample plates were automatically welded using a 6 kW fiber laser power source with a six-axis robotic welder. The filling wire was pure titanium Gr.2 with a diameter of 1.2 mm, which was fed by an automatic wire feeder. Before welding, the surfaces of the titanium plates were polished with sandpaper, and treated with NaOH and HCl solution to remove the oxide film on the surface. Then, the plates were rinsed and dried with alcohol. Autogenous laser welding and narrow-gap laser filling welding experiments were carried out to compare the welded morphology, microstructure and mechanical properties. During the laser welding process, a pure argon shielding device was designed and used to protect the welding pool area at high temperature above 100 ℃. Both the front and back weld zones were protected to avoid contamination from N, O and H elements in the air. The parameters were fixed in a way to achieve a defect-free weld bead with suitable weld morphology. Beam oscillation technology was used for narrow-gap laser welding to improve the sidewall fusion. The schematic diagram of the welding processes is shown in Figure 1. The plate gap for autogenous laser welding was set as 0.2 mm, whereas the gap distance was 4 mm for narrow-gap laser welding. The defocusing distances of the autogenous laser welding and narrow-gap laser welding were −2 mm and +5 mm, respectively [13,14].

After welding, the welded samples were transversely cut from the plate to measure the cross-section morphology. The corrosive liquid was a mixed solution of saprohydrofluoric acid and nitric acid with a ratio of 1:2 for 5 s. After corrosion, the morphologies and microstructure were obtained via optical microscope (XJZ-6A, Nanjiang Jiangnan Novel Optics Co., Ltd, Nanjing, China). The microhardness of the welded joint was measured with a Micro-Vickers machine (402 MVA, Wolpert Wilson Instruments, Norwood, MA, USA) with a load of 1.0 KN and the tensile test was carried out with a universal testing machine (SHT 4605, MTS, Shenzhen, China) with a tensile speed of 0.5 mm/min.

After butt welding, the metallographic sample was cut and corroded via Kroll reagent. The microstructure of the welded joint was analyzed via optical microscope, and the joint microhardness distribution was measured with to be a distance of 0.2 mm. The autogenously laser welded joint should meet the technical requirements of GB/T 37901-2019 [15] and the narrow-gap laser filling welded joint should meet the technical requirements of NB/T11270-2023 [16].

## 3. Results and Discussion

### 3.1. Bead Cross-Sectional Morphology and Weld Microstructure of Autogenous Laser Welding

**Figure 2:**
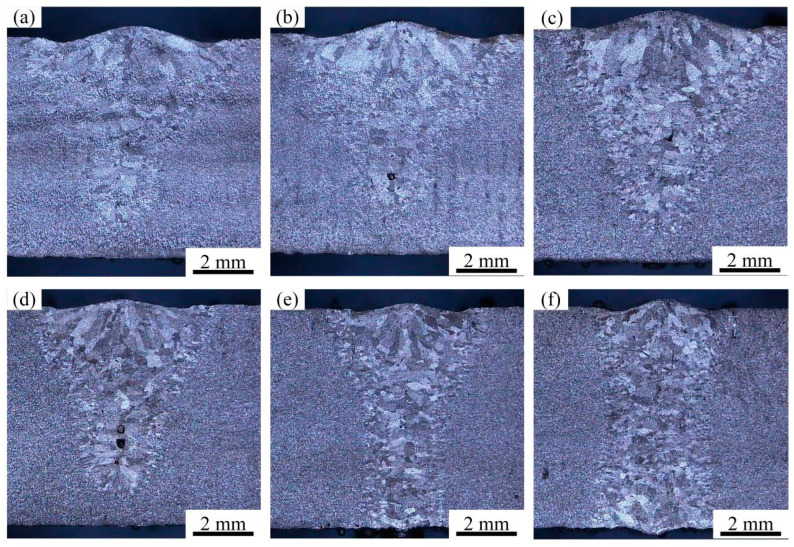
Bead morphology of the bead on plate autogenous laser welding: (**a**) 3300 W, 1.2 m/min; (**b**) 3360 W, 1.2 m/min; (**c**) 3420 W, 1.2 m/min; (**d**) 4020kW, 1.8 m/min; (**e**) 4080 W, 1.8 m/min; (**f**) 4800 W, 1.8 m/min;.

The weld morphologies of autogenous laser welding with various parameters are shown in Figure 2 and the weld dimensions are presented in Table 1. The requirement of the weld dimension was to ensure that the weld width was less than 5 mm in the case of full penetration. At the slower welding speed of 1.2 m/min, it can be seen that the weld width and penetration increased simultaneously with the increasing laser power. The weld cross section presented inverted triangle shapes with wider weld width and lower weld penetration. This was because the heat transferred in the direction of the melting width, which was greater than that in the direction of melting depth in the case of partial penetration. However, as the welding speed increased to 1.8 m/min and laser power increased to 4080 kW, the full penetration weld morphology was achieved. The laser keyhole can absorb more laser irradiated energy to increase weld penetration. The typical laser keyhole welding mode with funnel shape was obtained with a weld width of 3.2 mm. Furthermore, it was found from the weld cross-sectional morphologies that the weld porosities tended to form in a partial penetration weld, while nearly disappearing in a full penetration weld [17,18,19,20]. Some researchers have revealed that with the increased welding speed, weld porosities obviously reduced on account of the keyhole stability. During the full penetration welding process, the keyhole ran through the entire thickness direction and the plasma vapor was expelled from below the keyhole. It can be seen that the back of the welded zone had some spatters in full penetration mode, indicating the keyhole ran through the whole plate thickness and some liquid metal sprayed under the metal vapor. Therefore, some porosities produced by the unstable keyhole disappeared.

**Figure 3:**
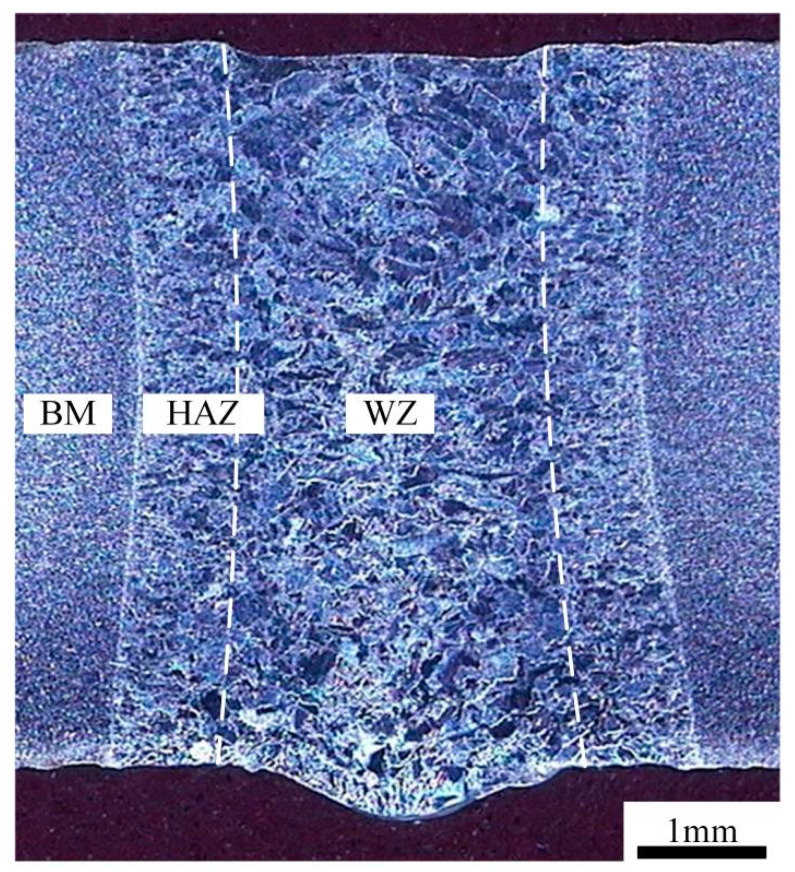
Butt bead morphology of the laser autogenously welded joint. (BM: base metal; HAZ: heat affected zone; WZ: weld zone; the white dotted line means fusion line).

The butt weld cross section of autogenous laser welding is shown in Figure 3. The weld was achieved with full penetration and lack of porosity or cracking. The welded joint was narrow in the middle zone and wider at the upper and lower surfaces. The middle zone was 4.06 mm and the width of the upper and lower surfaces was 4.31 mm and 4.80 mm, respectively. The upper surface with the slight undercut and the lower surface with the weld reinforcement were presented due to the gravity and fluidity of the molten pool. The macro appearance of the autogenous laser butt weld met the technical requirements of GB/T 37901-2019.

**Figure 4:**
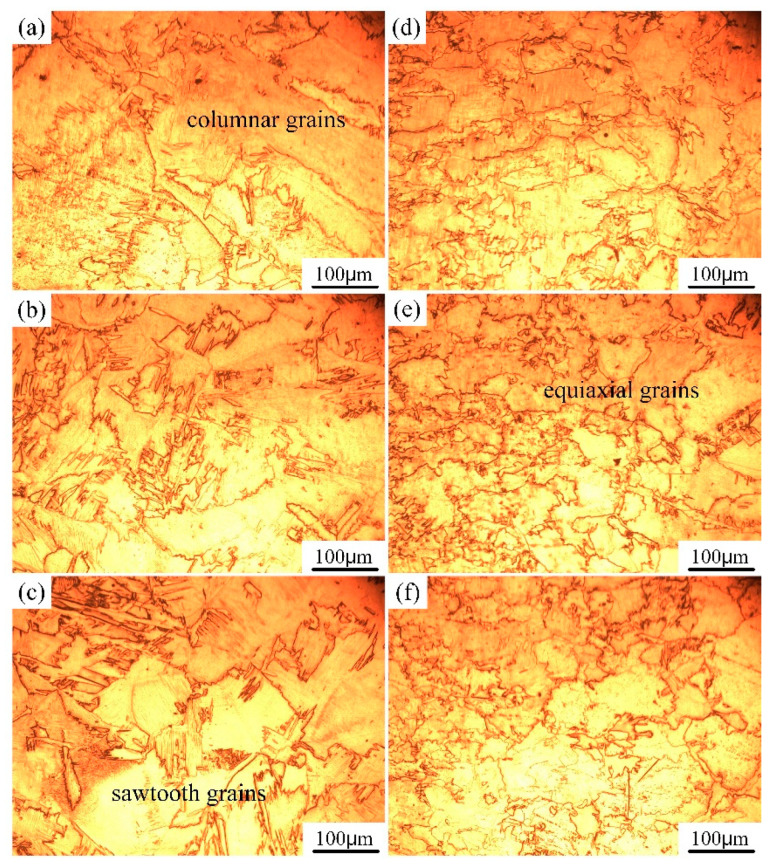
Microstructure of the laser autogenously welded joint in various zones: (**a**) upper zone, (**b**) middle zone, (**c**) lower zone of weld center, (**d**) upper zone, (**e**) middle zone, (**f**) lower zone of fusion line zone.

The macro morphology of autogenously laser welded joints is clearly divided into the base metal zone, the heat-affected zone and the fusion zone, as shown in Figure 4. As for the autogenous laser welding process, the concentrated laser energy and fast cooling rate led to a great temperature gradient, therefore, the microstructure in various zones of transverse and depth directions presented different characteristics. In the fusion line zone, the α grains showed irregular and various size, which was related to the cooling rate in the depth direction. The microstructure in the middle and lower zones showed equiaxial grain features, however, columnar grain morphology of a larger size were found in the upper zone. Moreover, it can be seen that the grains gradually grew from the heat-affected zone to the weld center zone. The grains in the heat-affected zone were relatively smaller than that in fusion zone, because this zone was mainly heated by the high temperature of molten pool. In the weld center, the relatively slower cooling rate and longer existing time of the molten pool provided a favorable condition for the growth of α grains. The columnar grains grew from two sides of the weld line to the weld center and had obvious growth direction. Part of the α grains existed in the form of irregular cross-arranged saw teeth. In the upper weld zone, the columnar grain was obviously greater because of the solidification essence of the weld molten pool. The upper weld zone had a smaller temperature gradient and longer existing time, which was beneficial to the grain growth.

### 3.2. Bead Cross-Sectional Morphology and Weld Microstructure of Narrow-Gap Laser Welding

**Figure 5:**
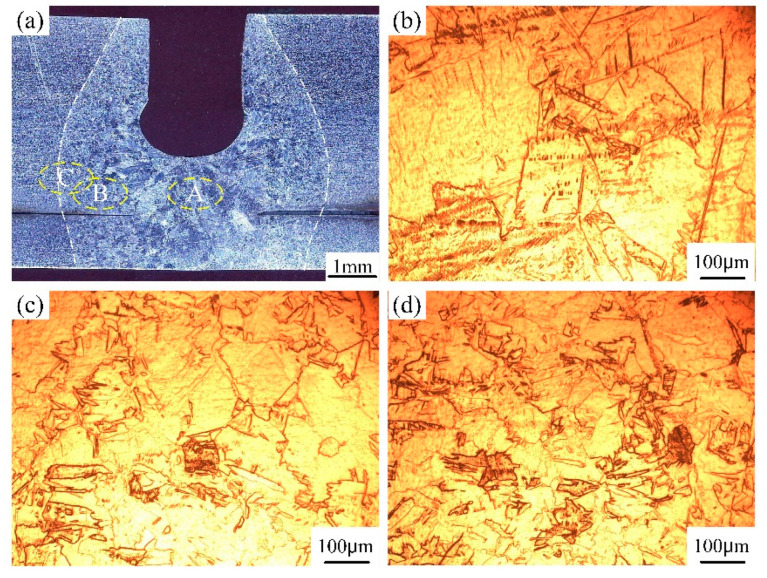
The weld cross-sectional morphology and microstructure in the first filling layer: (**a**) cross-sectional morphology; (**b**) microstructure in the weld center zone; (**c**) heat-affected zone close to the weld; (**d**) heat-affected zone close to the base metal. (A: WZ; B: fusion line zone; C: HAZ).

**Figure 6:**
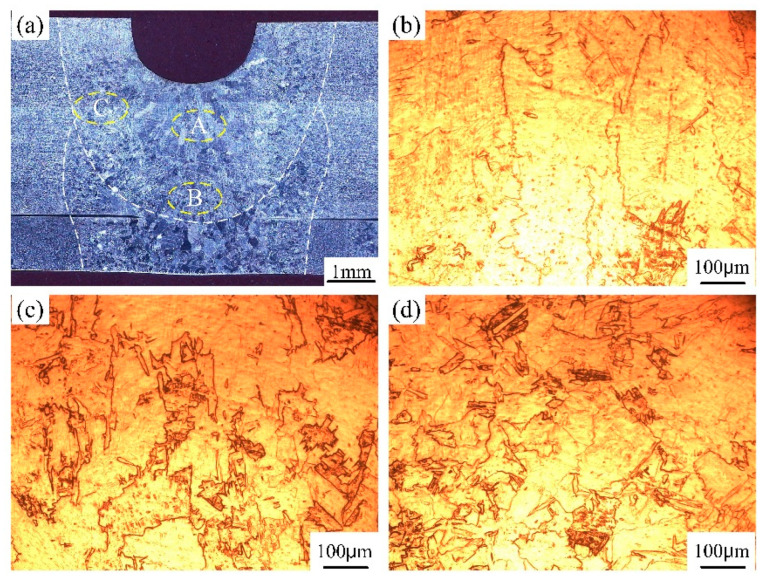
The weld cross-sectional morphology and microstructure in the second filling layer: (**a**) cross-sectional morphology; (**b**) microstructure in the central weld zone; (**c**) in the remelting zone; (**d**) in the heat-affected zone. (A: WZ; B: fusion line zone; C: HAZ).

**Figure 7:**
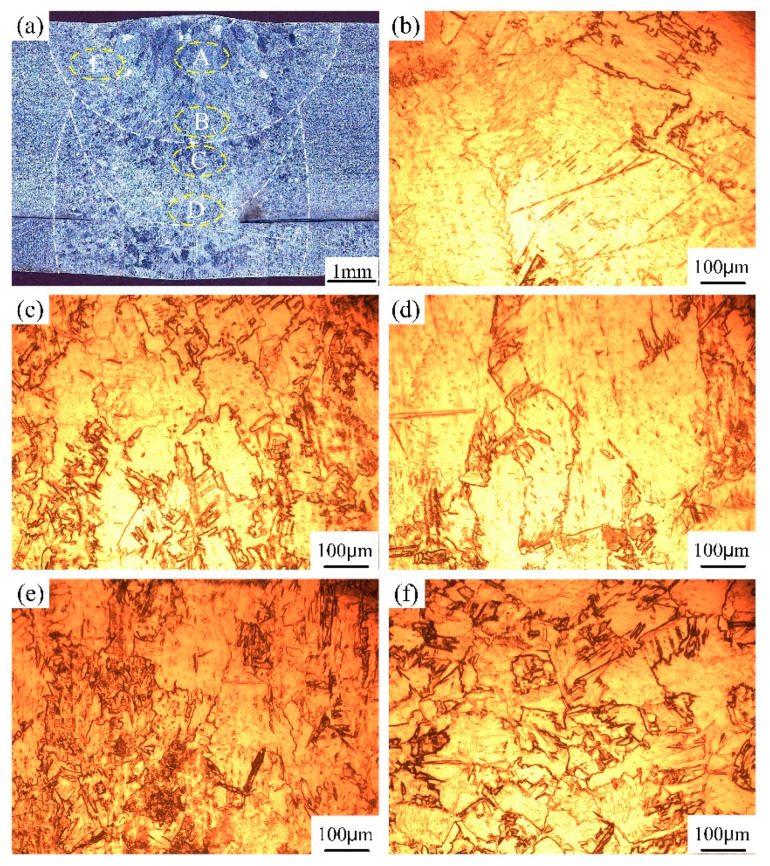
The weld cross-sectional morphology and microstructure in the third filling layer: (**a**) cross-sectional morphology; (**b**) amplifying image of zone A; (**c**) of zone B; (**d**) of zone C; (**e**) of zone D; (**f**) of zone E.

For the narrow-gap laser wire filling welding process of medium titanium plate, laser beam oscillation was used to solve the lack of fusion to the sidewall. Both the filling wire, groove sidewall and bottom base metal were melted by the irradiated laser energy, which was mixed and solidified to form the filling layer. The bead cross-sectional morphologies of each filling layer are shown in Figure 5a, Figure 6a and Figure 7a. The groove width and depth were 5 mm and 7 mm, respectively. The beam oscillation can reallocate laser energy into the groove from the Gaussian distribution mode to uniform distribution, especially increasing the laser energy on the sidewalls. It can be seen that the narrow-gap weld was completed by three filling layers without pores and incomplete fusion. However, the groove sidewalls were excessively melted and an undercut appeared in intermediate filling layers, which was due to the overallocation of laser energy to the sidewall via beam oscillation. The increase in the dilution rate of the base metal was not conducive to the improvement of joint performance for titanium alloy metal. Furthermore, during the multi-layer narrow-gap laser welding process, part of the former filling layer was also remelted by subsequent laser welding. The upper surface of the intermediate filling layers presented a smooth transition, indicating the better wetting between the filling material and the sidewalls.

The weld microstructure of each filling layer in various weld zones is presented in Figure 5, Figure 6 and Figure 7. The microstructure of laser filling welding is basically similar to the autogenous laser welding process, while some special characteristics, such as the grain feature and remelted microstructure, are presented. In the first filling layer, the weld filling metal was mainly composed of α columnar grains with massive size, which grew from the fusion line to the weld center. A small number of α acicular grains existed in the inner α columnar grains, as shown in Figure 5b–d. The heat-affected zone, fusion line and weld center underwent various welding heat cycles, resulting in great differences in grains feature. In the fusion line near to the weld zone and heat-affected zone, the α grains obviously grew with a serrated shape compared to the base metal. The isometric grain characteristics disappeared. The microstructure of the second and third filling layer is shown in Figure 6b–d and Figure 7b–f. The size of α columnar grains in the weld center was relatively larger than that of the first filling layer, which was attributed to the change in the heat dissipation mode. For the first and second filling layer, the laser input heat was transferred from the bottom and two sides of the groove, while the molten pool heat was only dissipated by the bottom of the groove. Therefore, the differences of solidification condition resulted in the weld microstructure diversity. Furthermore, after the completion of the second or third filling layer, the part of the first or second filling layer underwent a phase transformation and recrystallization process. The remelting zone between the filling layers experienced multiple thermal cycles. The original coarse α columnar grains underwent melting, solidification and microstructure transformation again during the welding process. The remelting zone had a better heat dissipation condition and comparatively faster cooling rate than the first melting process. Therefore, the grain size in the remelting zone of the first and second layers was finer with an irregular shape.

Compared with narrow-gap laser filling welding, autogenous laser welding has a concentrated laser energy, lower heat input, higher efficiency, narrower weld width and finer grain size. The highly irradiated power used in the laser keyhole welding mode caused the rapid heating, melting and evaporating of the plate surface. For the welding of thick plates, the three-dimensional heat dissipation model plays a role in microstructural evolution. With the continuous laser energy input, the keyhole was formed, leading to the laser-irradiated energy directly transferring in the depth direction. However, the heating energy only came from heat conduction outside the range of the heat source in the width direction. The temperature changed more dramatically along the width direction, resulting in the unevenly microstructural characteristics [3,21,22,23]. As for narrow-gap laser filling welding, the defocusing distance and beam oscillation led to a heat conduction welding mode, which produced a wider and shallower weld morphology. The heat dissipation model presented a two-dimensional model. The power density that the laser irradiated on the metal was lower and more uniform in the width direction because of the beam oscillation. The beam oscillation had a promotion effect on grain refinement by enhancing the liquidity of high-temperature liquid metal. Therefore, it can be seen that the grain size of the laser filling weld was similar to the autogenous laser welding except for the upper surface of the solidification zone.

### 3.3. Mechanical Properties Analysis of Welded Joints

**Figure 8:**
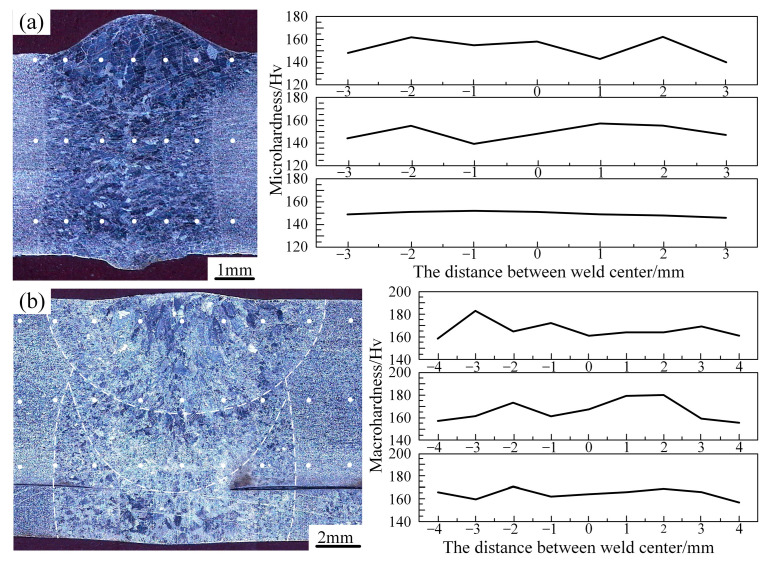
Microhardness distribution in weld cross section: (**a**) autogenously laser welding; (**b**) narrow-gap laser filling welding.

**Figure 9:**
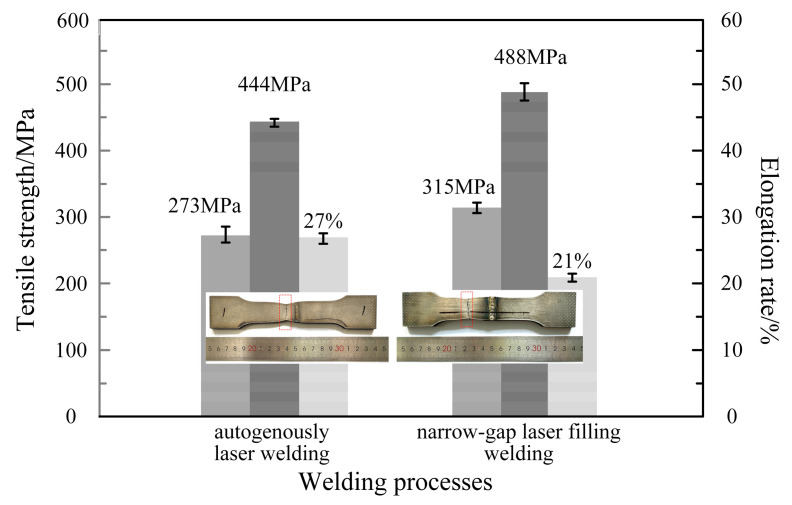
Tensile properties of welded joints at room temperature.

Figure 8 and Figure 9 show the microhardness distribution of the cross section, tensile strength and fracture sample of the laser welded joint. The autogenous laser welding and narrow-gap laser filling welding had a similar distribution trend in the base metal, heat-affected zone and weld fusion zone. The microhardness of the weld zone and the heat-affected zone was slightly higher than that of the base metal, and the microhardness reached its peak in the heat-affected zone. This was because of the existence of the needle and sawtooth α in the weld zone and heat-affected zone due to the unbalanced metallurgical process. In the heat-affected zone, the needle α phase was finer and denser, and the grain size was smaller than that in the welded zone. Some research revealed that the size and density of α grains affects the microhardness value [10], therefore, the microhardness value of the heat-affected zone near the weld was the highest in the entire weld. Both the autogenous laser and narrow-gap laser welded joints were fractured at the base metal zone from the fracture samples, indicating that the strength of the weld zone was higher than the base metal. The strength coefficient of the welded joint was greater than 1, meeting the requirements for a welded joint. However, it can be seen that the strength of the autogenously laser welded joint was lower and the elongation rate was higher than the narrow-gap laser welded joint, which was mainly because of the difference in width and strength of the welded zone. The autogenously laser welded joint had a narrower welded zone width, and the deformation during the tensile test was mainly focused on the base metal zone, which provided a better deformation behavior until fracture. However, for narrow-gap laser welding, the wider welding zone led to the base metal experiencing a larger deformation, causing a higher strength and lower elongation rate.

## 4. Conclusions

(1) Compared with autogenous laser and narrow-gap laser filling welding, the weld width of the narrow-gap laser welded joint was 12.5 mm, which was nearly three times than that of autogenous laser welding.
(2) The microstructure of the TA2 base metal was equiaxed α grains. In the weld zone of the autogenously laser and narrow-gap laser welded joints, the coarse columnar α grains grew from the fusion line, while in the heat-affected zone, the equiaxed α grains with needle and sawtooth α morphologies were presented. The grain size of autogenous laser welding was obviously smaller and uniform in depth direction than that of narrow-gap laser welding.
(3) The weld microhardness and tensile strength of autogenous laser and narrow-gap laser welding had common characteristics. The heat-affected zone had the maximum weld microhardness due to the denser α grains with needle morphologies. The tensile samples all fractured at the base metal, indicating the welded joint strength efficiency was greater than 1.

## Figures and Tables

**Table 1:** The detailed weld dimensions with various welding parameters.

No.	Laser Power	Velocity	Upper Surface Width	Weld Penetration	Reinforcement	Weld Width in 2/4 Penetration
	W	m/min	mm	mm	mm	mm
a	3300	1.2	4.9	5.1	0.3	1.8
b	3360	1.2	5.2	4.8	0.4	1.3
c	3420	1.2	5.1	5.1	0.4	1.7
d	4020	1.8	3.7	4.8	0.2	1.5
e	4080	1.8	3.2	6.0	0.1	/
f	4800	1.8	3.3	6.0	0.2	/

## Data Availability

The original contributions presented in the study are included in the article, further inquiries can be directed to the corresponding author.
